# Tonsilar NK Cells Restrict B Cell Transformation by the Epstein-Barr Virus via IFN-γ

**DOI:** 10.1371/journal.ppat.0040027

**Published:** 2008-02-08

**Authors:** Till Strowig, Fabienne Brilot, Frida Arrey, Gwenola Bougras, Dolca Thomas, William A Muller, Christian Münz

**Affiliations:** 1 Laboratory of Viral Immunobiology, The Rockefeller University, New York, New York, United States of America; 2 Christopher H. Browne Center for Immunology and Immune Diseases, The Rockefeller University, New York, New York, United States of America; 3 Department of Nephrology, Weill Medical College, Cornell University, New York, New York, United States of America; 4 Department of Medicine, Weill Medical College, Cornell University, New York, New York, United States of America; 5 Department of Pathology and Laboratory Medicine, Weill Medical College, Cornell University, New York, New York, United States of America; Mount Sinai School of Medicine, United States of America

## Abstract

Cells of the innate immune system act in synergy to provide a first line of defense against pathogens. Here we describe that dendritic cells (DCs), matured with viral products or mimics thereof, including Epstein-Barr virus (EBV), activated natural killer (NK) cells more efficiently than other mature DC preparations. CD56^bright^CD16^−^ NK cells, which are enriched in human secondary lymphoid tissues, responded primarily to this DC activation. DCs elicited 50-fold stronger interferon-γ (IFN-γ) secretion from tonsilar NK cells than from peripheral blood NK cells, reaching levels that inhibited B cell transformation by EBV. In fact, 100- to 1,000-fold less tonsilar than peripheral blood NK cells were required to achieve the same protection in vitro, indicating that innate immune control of EBV by NK cells is most efficient at this primary site of EBV infection. The high IFN-γ concentrations, produced by tonsilar NK cells, delayed latent EBV antigen expression, resulting in decreased B cell proliferation during the first week after EBV infection in vitro. These results suggest that NK cell activation by DCs can limit primary EBV infection in tonsils until adaptive immunity establishes immune control of this persistent and oncogenic human pathogen.

## Author Summary

Epstein-Barr virus (EBV) establishes a persistent infection in nearly all human adults. Due to its tumor causing potential EBV infection has to be continuously controlled by the immune system in virus carriers. We demonstrate here that in the first week after infection, when other EBV-specific immune responses are still being recruited, human natural killer (NK) cells are able to prevent transformation of the main host cell type by EBV, the human B cell. Especially NK cells of tonsils, the primary site of EBV infection, inhibit B cell transformation by EBV after they have been activated by dendritic cells (DCs). For this protective function, EBV can directly stimulate DCs to efficiently activate NK cells. Interestingly, NK cells primarily prevent B cell transformation by EBV via secretion of the anti-viral cytokine IFN-γ, and NK cells from tonsils and lymph nodes produce 5-fold more of this cytokine than their peripheral blood counterparts. These data suggest that specialized NK cells in tonsils, the mucosal entry site of EBV, can be efficiently stimulated by EBV-activated DCs, and then limit EBV-induced B cell transformation until EBV-specific immune control by other components of the immune system is established.

## Introduction

Epstein-Barr (EBV) is a lymphotropic γ-herpes virus infecting over 90% of the human adult population [1,2]. A striking feature that the virus shares with other γ-herpes viruses is its oncogenic potential. This transforming property can be observed in vitro and in vivo both in immunocompetent and more frequently in immunosuppressed individuals. In the latter group, EBV causes tumors such as post-transplant lymphoproliferative disease and immunoblastic lymphoma, whereas, nasopharyngeal carcinoma, Hodgkin's disease and endemic Burkitt's lymphoma are the most prominent EBV-associated malignancies in immunocompetent individuals [3]. However, in most individuals, the lifelong chronic infection with EBV is fortunately free of complications due to effective immune control, primarily mediated by CD4^+^ and CD8^+^ T cells [4]. In addition to protective T cell immunity in healthy virus carriers, several lines of evidence suggest a role for innate lymphocytes in the resistance against EBV-associated malignancies. Firstly, in male patients with X-linked lymphoproliferative disease (XLP), who frequently succumb after primary EBV infection to EBV-induced lymphomas, a mutation in the SAP gene leads to defective recognition of EBV-transformed B cells by NK cells [5–7]. While SAP mutations not only affect NK cell function, this defective recognition most likely contributes to loss of EBV-specific immune control. Secondly, IL-2-activated peripheral blood NK cells have been shown to restrict EBV-induced B cell transformation in vitro [8–10]. Thirdly, NK cell depletion from PBMCs prior to adoptive transfer into SCID mice, rendered the animals more susceptible to tumor development after transfer of EBV-transformed B cells [11]. Fourthly, activated NK cells have been shown to lyse lytically EBV replicating B cells [12]. Fifthly, a novel primary immunodeficiency with a specific NK cell defect was recently reported to be associated with EBV-driven lymphoproliferative disease [13]. Therefore, NK cells may be involved in the early phase of the EBV-specific immune response.

NK cells are innate lymphocytes that play an important role in the control of infections and the immune surveillance of tumors [14]. In particular, early after primary viral infections they are thought to limit the viral burden until virus-specific T cells are able to eliminate the infection or control viral titers at low levels [15]. During infection with the β-herpes virus murine cytomegalovirus (MCMV), NK cells have been shown to be crucial for limiting viral replication [16]. Indeed, lack of the activating NK cell receptor Ly49H, involved in the recognition of the MCMV m157 protein, confers susceptibility to fatal MCMV infection in BALB/c mice, whereas Ly49H-expressing C57BL/6 mice survive infection with high viral titers [17]. Nonetheless, NK cells need to receive additional signals to successfully protect against MCMV infection and these are provided by DCs [18–22]. Notably, NK cells produce cytokines such as IFN-γ, proliferate and increase their cytotoxicity upon activation by both myeloid and plasmacytoid DCs [23]. Therefore, DCs seem to activate NK cells early after infection in order to restrict pathogen replication until the adaptive immune system establishes long-lasting immune control.

Although there are significant similarities between murine and human NK cell activation by DCs, human and mouse NK cell phenotypes and functions differ substantially and these differences also influence NK cell/DC interactions. Two main functional NK cell subsets have been distinguished in humans: CD56^dim^CD16^+^ NK cells readily lyse susceptible target cells, but secrete only low levels of cytokines after activation. In contrast, CD56^bright^CD16^−^ NK cells produce large amounts of cytokines upon stimulation and acquire cytotoxicity only after prolonged activation [24,25]. Around 90% of human peripheral blood NK cells are CD56^dim^CD16^+^, whereas CD56^bright^CD16^−^ NK cells constitute less than 10% of the peripheral blood NK cell pool. However, in secondary lymphoid organs such as tonsils and lymph nodes, the CD56^bright^CD16^−^ NK cells are the dominant subset [26–28]. Interestingly, human DCs primarily activate this NK cell subset [29,30]. Because the tonsils are the primary site of EBV infection, we investigated whether the DC/NK cell crosstalk could trigger NK cells to limit B cell transformation during EBV infection.

We show here that human NK cells from peripheral blood, spleen, and tonsils limit the outgrowth of EBV-infected B cells after activation by DCs in vitro. Monocyte-derived DCs, which were matured with viral double-stranded RNA or its analog polyinosine-polycytidylic acid (polyI:C) to produce high levels of IL-12, stimulated strong IFN-γ secretion and proliferation of mainly the CD56^bright^CD16^−^ NK cell subset. These NK cells then significant restricted B cell transformation by EBV. Tonsilar NK cells were more efficient in inhibiting EBV-induced B cell transformation in vitro than peripheral blood NK cells and secreted high IFN-γ levels, which proved sufficient to limit B cell-transformation despite their low frequency at the site of primary infection. Restriction of B cell transformation by EBV was primarily due to IFN-γ secretion by DC-activated NK cells, and due to regulation of the EBV latency program by this cytokine. Interestingly, myeloid DCs, matured by exposure to EBV, were also able to elicit IFN-γ secretion by NK cells to levels protective against EBV-induced B cell transformation. Contrary to the original hypothesis that NK cells control pathogens via spontaneous cytotoxicity, which gave rise to the name of this innate lymphocyte subset, we demonstrate that these NK cell responses require activation by DCs and are mediated by cytokines. These data provide the first evidence for a direct antiviral effector function of NK cells in secondary lymphoid tissues, which might limit EBV infection until the adaptive immune system efficiently controls it.

## Results

### NK Cell Activation by Differently Matured DCs

**Figure 1:**
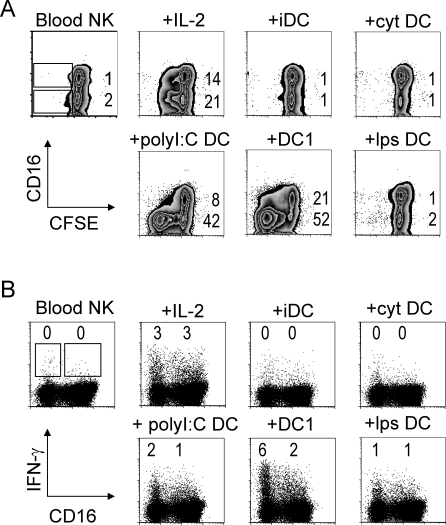
DCs Activate NK Cells Most Efficiently after Maturation with the dsRNA Analog polyI:C Differently matured DCs were compared to immature DCs for their ability to induce peripheral blood NK cell proliferation (A) and IFN-γ secretion (B). The tested maturation stimuli included (i) the standard proinflammatory cytokine cocktail consisting of IL-1β, IL-6, TNF-α, and prostaglandin E_2_ (cyt DC); (ii) the TLR3 and mda-5 agonist polyI:C (polyI:C DC); (iii) a cocktail of inflammatory cytokines (IL-1β, TNF-α IFN-α, IFN-γ) and polyI:C (DC1); (iv) the TLR4 agonist LPS (lps DC). These differently matured DCs were compared to immature DCs (iDC) and stimulation with IL-2 alone. (A) CFSE-labeled NK cells and DCs were cultured for 6d at a ratio of 5:1, and proliferation was analyzed by gating on CD3^−^CD56^+^ cells. Percentages of CFSE dilute CD16^+^ and CD16^−^ NK cells are indicated. (B) NK cells and DCs were cultured for 20 h at a ratio of 2:1 and BFA was added for the last 8 h. IFN-γ production of CD3^−^CD56^+^ cells was analyzed. Percentages of IFN-γ positive NK cells are indicated. Similar results were obtained in three independent experiments.

The outcome of the crosstalk between NK cells and DCs depends strongly on the activation status of both cell types [27]. We investigated how differently matured human monocyte-derived DCs vary in their capacity to elicit proliferation and IFN-γ secretion by NK cells in order to define the optimal activation conditions for anti-viral NK cell responses. DCs were matured using a standard mixture of proinflammatory cytokines (IL-1β, IL-6, TNF-α and PGE_2_: cyt DC), the TLR3 and mda-5 ligand polyI:C (polyI:C DC), and the TLR4 ligand LPS (lps DC). In addition, DCs were matured with poly I:C supplemented with proinflammatory cytokines (IL-1β and TNF-α) and type I and II interferons (DC1) to generate DC1 cells according to Mailliard and colleagues [31]. As expected, all of these maturation stimuli were able to significantly up-regulate MHC class II and costimulatory molecules such as CD80, CD83 and CD86 (Table S1). However, when we compared different DC preparations for their capacity to activate NK cells from peripheral blood, we found that DCs matured with polyI:C were far superior in NK cell activation compared to the other DC preparations (Figure 1A). polyI:C DCs and DC1s induced strong proliferation with 40–75% of the NK cells cycling after 6 days. Further characterization showed that the number of NK cells producing IFN-γ increased significantly (4-fold to 10-fold), when they were activated by polyI:C DCs and DC1s compared to immature DCs (iDCs) or cyt DCs, respectively (Figure 1B). In line with previous findings, the CD56^bright^CD16^−^ NK cells were preferentially stimulated by these DC preparations to proliferate and secrete IFN-γ (Figure 1). These data demonstrate that DCs matured by polyI:C are efficient stimulators of CD56^bright^ CD16^−^ NK cells, the NK cell subset enriched in human secondary lymphoid tissues.

### Production of NK Cell Stimulatory Cytokines upon DC Maturation with Viral Stimuli

To characterize the mechanism of DC-mediated NK activation, we compared the production of NK cell stimulatory cytokines by DCs after maturation. Previous work indicated important roles for IL-12, IL-15, and IL-18 in the activation of NK cell proliferation and IFN-γ secretion [28,32]. We detected only little (<50 pg/ml) or no secretion of the bioactive form of IL-12 (IL-12p70) by cyt DCs or iDCs, respectively, whereas polyI:C DCs and DC1s produced high amounts of this cytokine (up to 5000 pg/ml) (Table 1). LPS-matured DCs produced intermediate amounts of IL-12p70. Monocyte-derived DCs matured with EBV-derived dsRNA produced slightly higher IL-12 levels than LPS matured DCs (up to 200pg/ml). Addition of recombinant IL-12p70 at comparable levels (≥100pg/ml) induced both proliferation and IFN-γ production by NK cells (data not shown). IL-15 secretion and surface expression of IL-15 and IL-15Rα were also primarily induced by incubation of DCs with polyI:C (Tables 1 and S1). IL-18 secretion was not detectable for all DC preparations tested (Table 1). In line with these observations, IFN-γ secretion of NK cells stimulated by DC1s was mainly dependent on IL-12 (80%, *p* < 0.01) and to a lesser degree on IL-18 (15%, *p* < 0.05) (Figure S1A). Similar results were obtained with polyI:C DCs (data not shown). Consistent with the hypothesis that polyI:C-matured DCs mainly elicited IFN-γ secretion of NK cells via their high levels of secreted IL-12, this NK cell/DC interaction was not sensitive to transwell separation (Figure S1B). Furthermore, we found that NK cell proliferation upon coculture with DC1s (Figure S1C) and polyI:C DCs (data not shown) could be blocked with an IL-12 specific antibody by 90% or 80%, respectively (*p* < 0.01 for both), and again transwell experiments showed that direct cell contact was not required (data not shown). Blocking of IL-15 also significantly decreased numbers of surviving NK cells when combined with anti-IL-12 antibodies (*p* < 0.01), however blocking of IL-15 or IL-18 alone did not significantly decrease survival (Figure S1D). In addition, antibody blocking of IL-2 did not influence DC induced proliferation and IFN-γ production by NK cells (Figure S2). These data suggest that polyI:C and EBV-derived dsRNA elicit IL-12 production, which in turn stimulates NK cell proliferation and IFN-γ production by NK cells.

**Table 1:**
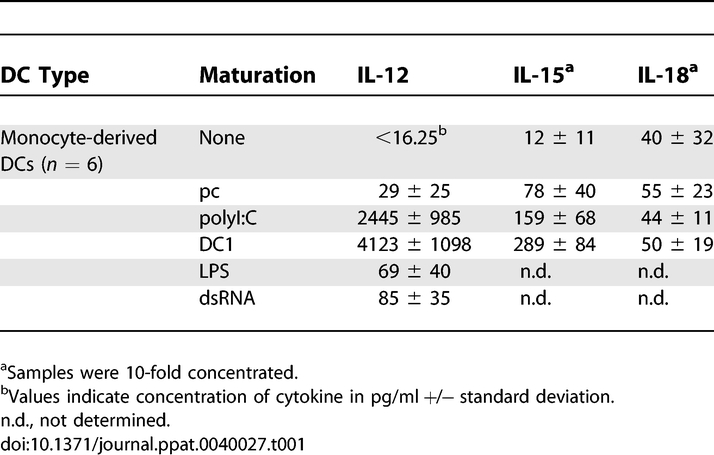
Secretion of IL-12, IL-15, and IL-18 by DCs

### Restriction of EBV-induced B Cell Transformation by NK Cells upon Activation by polyI:C Matured DCs

**Figure 2:**
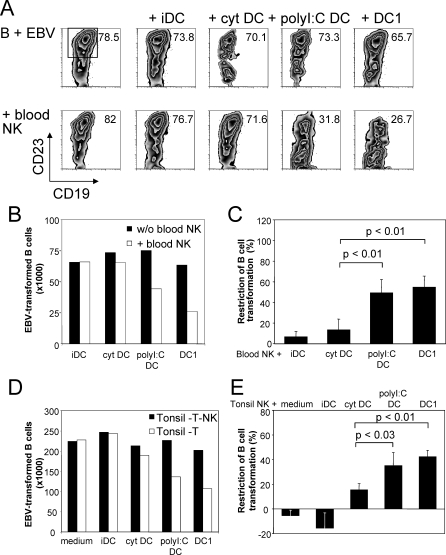
NK Cells from Blood and Tonsil Activated by PolyI:C-matured DCs Limit EBV-mediated B Cell Transformation (A) B cells were infected with EBV and cultured for 12 d alone, with DCs or NK cells and with DCs plus NK cells. The indicated percentage of B cell transformation was evaluated by determining the percentage of transformed CD19^+^CD23^+^B cells within gated CD19^+^CD21^+^ B cells. (B and C) Total numbers of transformed B cells were determined from live cell numbers and the percentage of transformed CD19^+^CD23^+^B cells in the different cultures. Total transformed B cell numbers for one representative experiment (B), and differences in total transformed B cell numbers for all experiments (percentage restriction of B cell transformation) (C), were analyzed with or without peripheral blood NK cell addition for the indicated DC maturation conditions. Data represent results from eight independent experiments (mean ± standard error of the mean). (D and E) Mononuclear cells from tonsils were depleted of CD3^+^ T cells or CD3^+^ T and CD56^+^ NK cells. Total numbers of transformed B cells were determined from live cell numbers and the percentage of transformed CD19^+^CD23^+^ B cells in the different cultures. Total transformed B cell numbers for one representative experiment (D), and differences in total transformed B cell numbers for all experiments (percentage restriction of B cell transformation) (E), were analyzed with and without tonsilar NK cells for the indicated DC maturation conditions. Data represent results from six independent experiments (mean ± standard error of the mean).

In contrast to previous studies focusing on IL-2 activation of NK cells, we addressed the question whether NK cells restrict EBV-induced B cell transformation after activation by cells of the innate immune system. Because interactions between NK cells and DCs have been shown to be essential for virus control in murine models of herpes virus infections [19,33,34], we specifically investigated whether DCs can activate NK cells to limit EBV-mediated B cell transformation. For these experiments we used monocyte-derived DCs, since they can be generated at sufficient numbers to allow functional experiments. When we infected purified B cells with EBV and cocultured them with resting purified peripheral blood NK cells at ratios of 5:1 (NK to B cells) we could not observe any restriction of B cell transformation. Similarly, addition of iDCs or cyt DCs induced only limited NK cell-mediated inhibition of EBV-transformed B cell outgrowth (6 or 13%, respectively) (Figure 2A–2C). However, in cultures with NK cells and polyI:C DCs or DC1s, we observed a 49 or 55% (both *p* < 0.01) reduction of the number of transformed B cells, respectively (Figure 2B and 2C). The DC preparations used in this study had no significant direct effect on B cell transformation by EBV (data not shown). This demonstrated that DC-activated NK cells can inhibit B cell transformation by EBV.

### Low Numbers of Tonsilar NK Cells Restrict EBV-Induced B Cell Transformation

Tonsils are the primary infection sites for EBV and harbor enriched populations of CD56^bright^CD16^−^ NK cells, which can be efficiently activated by DCs. Therefore, we investigated whether tonsilar NK cells can restrict EBV-induced B cell transformation. For this purpose, we depleted tonsilar mononuclear cells of CD3^+^ T cells by cell sorting and compared the numbers of transformed B cells after EBV infection to cultures that were depleted of both CD3^+^ T and CD56^+^ NK cells (Figure 2D and 2E). Without addition of DCs and with the addition of allogeneic iDCs or cyt DCs, we did not observe any significant difference in the number of transformed B cells after 12 days (Figure 2D and 2E). However, we observed a 35% or 42% (*p* < 0.03 and *p* < 0.01) reduction of the number of transformed B cells after addition of allogeneic polyI:C DCs or DC1s, respectively (Figure 2D and 2E). These data suggest that tonsilar NK cells are able to restrict B cell transformation by EBV after stimulation by polyI:C DCs and DC1s.

**Figure 3:**
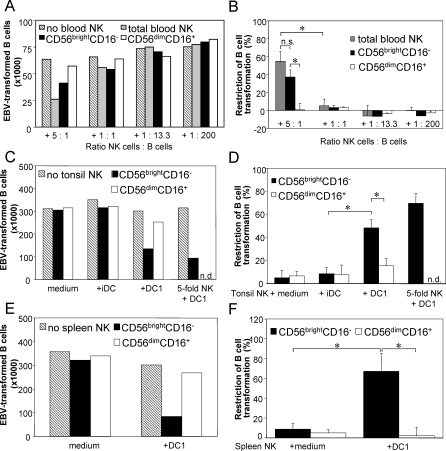
CD56^bright^CD16^−^ Cells from Blood, Tonsil, and Spleen Limit EBV-mediated B Cell Transformation after Activation by DCs (A and B) B cells were infected with EBV and cultured for 12 d with peripheral blood NK cells and DC1s at the indicated NK cell to B cell ratios. Total NK cells were compared to sorted CD56^bright^CD16^−^ and CD56^dim^CD16^+^ NK cells in their ability to limit EBV-mediated B cell transformation. Total transformed B cell numbers for one representative experiment (A) and restriction of B cell transformation for all experiments (B) were compared after addition of unseparated bulk NK cells (total NK), CD56^dim^CD16^+^ NK cells (90% of the bulk NK cell number) or CD56^bright^CD16^−^ NK cells (10% of the bulk NK cell number, to respect the NK subset distribution in peripheral blood) (*, *p* < 0.01). (C and D) Tonsilar B cells were infected with EBV and cultured for 12 d alone or with autologous tonsilar NK cell subsets at 2 ratios (NK, 5,000 and 5-fold NK, 25,000) in the absence or presence of allogeneic iDCs or DC1s (*, *p* < 0.01). Total transformed B cell numbers for one representative experiment (C), and restriction of B cell transformation for all experiments are shown (D). (E and F) Splenic B cells were infected with EBV and cultured for 12 d alone or with autologous splenic NK cell subsets in the absence or presence of autologous splenic DCs matured with polyI:C, TNF-α, IL-1β, IFN-α and IFN-γ (*, *p* < 0.01). Total transformed B cell numbers for one representative experiment (E), and restriction of B cell transformation for all experiments are shown (F). Data in (A–F) represent results from three independent experiments (mean ± standard error of the mean).

NK cells are present in tonsils at lower frequencies than in peripheral blood (0.3% compared to 10%), corresponding to only 3000 NK cells in a tonsilar B cell transformation assay with 6 × 10^5^ B cells (NK to B cell ratio of 1:200). However, the ratio between CD56^dim^CD16^+^ and CD56^bright^CD16^−^ NK cells is almost reversed between these organs with 75% of all NK cells being CD56^bright^CD16^−^ in tonsils and only 5% being CD56^bright^CD16^−^ in peripheral blood [27]. To compare directly the abilities of NK cells from blood and tonsils to restrict B cell transformation, we tested different ratios of blood NK cells to B cells. In addition to an intermediate ratio (NK:B = 1:1), we mimicked NK cell to B cell ratios found in tonsils (NK:B = 1:200; 60% B and 0.3% NK cells). We also added sufficient peripheral blood NK cells to mimic the CD56^bright^CD16^−^ NK cell to B cell ratio found in tonsil (NK:B = 1:13.3; CD56^bright^CD16^−^ NK cells are 15fold enriched in bulk tonsilar NK cells compared to blood NK cells: 75% in tonsil and 5% in blood). Already at a ratio of 1:1 we could only detect a limited 6% reduction of B cell transformation compared to controls (Figure 3A and 3B). These findings indicate that tonsilar NK cells are 1000-fold more efficient in controlling B cell transformation than peripheral blood NK cells.

### CD56^bright^CD16^−^ NK Cells Restrict EBV-Induced B Cell Transformation Most Efficiently

To study which subset of NK cells limits B cell transformation, we sorted NK cell subsets from blood and activated them with DC1s. While sorted CD56^dim^CD16^+^ NK cells did not mediate restriction of B cell transformation (NK:B = 4.5:1), low numbers of CD56^bright^CD16^−^ NK cells (NK:B = 1:2) were found to inhibit B cell transformation (34%) similar to 10-fold higher numbers of bulk NK cells (Figure 3A and 3B). Next, we sorted NK cell subsets from tonsils and observed that again CD56^bright^CD16^−^ NK cells but not CD56^dim^CD16^+^ NK cells were efficiently limiting B cell transformation after activation by DC1 (Figure 3C and 3D). At 10-fold lower numbers, tonsilar CD56^bright^CD16^−^ cells inhibited B cell transformation more (48.3% vs. 34%) than their counterparts in blood. Since CD56^bright^CD16^−^ NK cells are 15-fold enriched in tonsils compared to blood, NK cell mediated restriction is achieved with at least 150-fold lower bulk NK cell numbers in this organ. Further increasing the number of tonsilar NK cells 5-fold (NK:B = 1:40) led to restriction of B cell transformation by 69.7% (Figure 3C and 3D). Moreover, high numbers of splenic CD56^bright^CD16^−^ NK cells (NK:B = 5:1), a mixture of blood and secondary lymphoid organ NK cells, when activated with matured autologous myeloid CD11c^high^ DCs isolated from spleen, restricted B cell transformation by 67% (Figure 3E and 3F). Since we observed that at lower ratios of blood NK cells to B cells, even purified CD56^bright^CD16^−^ peripheral blood NK cells were unable to limit B cell transformation after activation by polyI:C DCs and DC1s, whereas tonsilar NK cells were still able to restrict EBV-induced B cell transformation, we concluded that tonsilar CD56^bright^CD16^−^ NK cells are functionally different from their counterparts in peripheral blood, and inhibit EBV-induced B cell transformation more efficiently.

### Tonsilar and Lymph Node NK Cells Produce Higher Levels of the Anti-viral Cytokine IFN-γ than Peripheral Blood NK Cells

**Figure 4:**
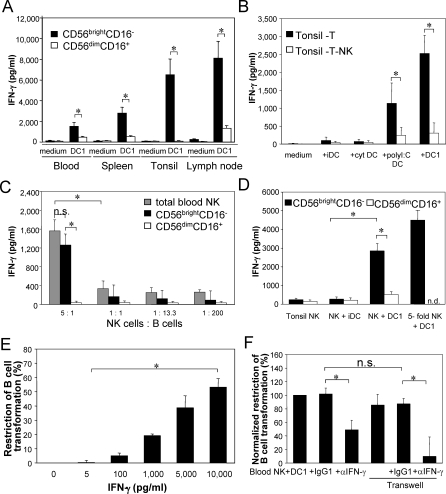
IFN-γ Secreted by NK Cells Restricts EBV-mediated B Cell Transformation (A) Sorted NK cell subsets from blood, spleen, tonsil, and lymph node were cultured alone or with DC1s, and IFN-γ levels were quantified by ELISA after 20 h. (B) IFN-γ secreted by tonsilar NK cells after activation by differently matured DCs was detected in supernatants of B cell transformation assays by ELISA. (Tonsil–T, tonsilar cultures depleted of T cells; Tonsil–T-NK, tonsilar cultures depleted of T and NK cells.) (C) IFN-γ secreted by peripheral blood NK cells and NK cell subsets was detected in supernatants of B cell transformation assays by ELISA. (D) IFN-γ secreted by purified tonsilar NK cell subsets was detected in supernatants of B cell transformation assays by ELISA. (E) Peripheral blood B cells were infected with EBV and increasing concentrations of IFN-γ were added. Restriction of B cell transformation was analyzed after 12 d by comparing numbers of transformed B cells with and without IFN-γ. (F) Blocking antibodies against IFN-γ were added to B cell transformation assays with B cells, NK cells, and DC1s from peripheral blood. Where indicated, NK cells were separated from B cells and DCs by transwell membranes. Results from at least three independent experiments were summarized (mean ± standard deviation) (*, *p* < 0.03).

Human NK cells from secondary lymphoid organs such as tonsils produce IFN-γ rapidly upon activation and this antiviral cytokine contributes directly to control early infection in murine models of herpes virus infection [33]. When we compared the production of IFN-γ upon NK cell/DC coculture, we observed that NK cells from tonsil and lymph node produced significantly more IFN-γ than their equivalents from blood or spleen (Figure 4A). Comparing CD56^bright^CD16^−^ NK cells, tonsilar and lymph node cells produced 5-fold more IFN-γ than peripheral blood cells, which amounted to a 50-fold difference when bulk NK cell cultures were analyzed due to the enrichment of CD56^bright^CD16^−^ NK cells in these organs. It had recently been reported that IL-18 exposed blood NK cells develop into a CD56^bright^CD83^+^CCR7^+^ NK cell subset with superior IFN-γ production [35]. In order to test if an enrichment of this NK cell subset could account for the superior ability of tonsilar NK cells to produce IFN-γ, we analyzed CD83 and CCR7 expression on tonsilar NK cells (Figure S4). Confirming our previously published data [28], we found no CCR7 expression on tonsilar NK cells, and only a minor population expressed CD83. Therefore, an enrichment of CD83^+^CCR7^+^ NK cells with superior IFN-γ production does not explain why NK cells from secondary lymphoid organs produce more IFN-γ than their peripheral blood counterparts.

### IFN-γ Levels Correlate with NK Cell Induced Restriction of B Cell Transformation by EBV

Higher IFN-γ secretion by tonsilar NK cells was also apparent when we then quantified the levels of IFN-γ in the culture supernatants of the B cell transformation assay after 12 days. We detected high levels in cocultures of NK cells from blood with polyI:C DCs (960 pg/ml; data not shown) or DC1s (1560 pg/ml; Figure 4C), but only with the highest numbers of NK cells (NK:B = 5:1). However, IFN-γ levels were even higher in B cell transformation cultures with bulk tonsilar NK cells and polyI:C DCs (1140 pg/ml) or DC1s (2500 pg/ml), and this IFN-γ secretion was NK cell dependent (Figure 4B). Furthermore, we detected similar levels of IFN-γ also in cultures with sorted CD56^bright^CD16^−^ NK cells from blood, tonsil and spleen (Figure 4C and 4D and data not shown), reaching up to 4000pg/ml IFN-γ concentrations with purified tonsilar CD56^bright^CD16^−^ NK cells. Therefore, only DC/NK cell co-cultures with polyI:C matured DCs and either high peripheral blood or low tonsilar NK cell numbers produce IFN-γ concentrations above 1000 pg/ml, and only these high IFN-γ levels correlate with control of EBV-transformed B cells.

### Restriction of EBV-Induced B Cell Transformation by NK Cells Relies on IFN-γ

In order to estimate the contribution of NK cell-produced IFN-γ on control of EBV-infected B cells, we added recombinant IFN-γ to purified and EBV-infected B cells from blood, spleen and tonsil. We detected fewer transformed B cells in cultures with high levels of IFN-γ (from 1000 to 10000 pg/ml) compared to controls with low levels (from 10 pg/ml to 200 pg/ml) of IFN-γ or without IFN-γ (Figure 4E). Indeed, 42% restriction of B cell transformation (*p* < 0.02) was observed in tonsilar B cell cultures with 5000 pg/ml IFN-γ, an IFN-γ concentration that was produced by tonsilar NK cells upon culture with polyI:C DCs and DC1s, and similar to measured IFN-γ levels in tonsil cell cultures (Figure 4A and 4D). However, IFN-γ mediated restriction of B cell transformation by EBV only limits, but does not eradicate EBV infection, since even with IFN-γ concentrations exceeding 10,000 pg/ml, we never observed more than 80% inhibition of B cell transformation by EBV (data not shown). Finally, DC activated NK cell were still able to mediate restriction when separated by transwell from EBV-infected B cells, and we could block inhibition of B cell transformation by over 60% using blocking antibodies against IFN-γ (Figure 4F). Therefore, IFN-γ contributes to NK cell-mediated restriction of EBV-induced B cell transformation.

### Myeloid DCs Sense EBV Directly and Elicit IFN-γ Secretion by NK Cells to Levels Protective against EBV-Induced B Cell Transformation

**Figure 5:**
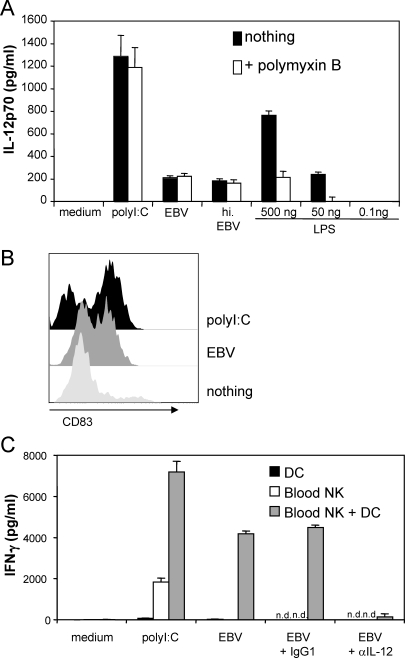
Myeloid DCs Can Sense EBV and Subsequently Activate NK Cells via IL-12 (A) CD11c^+^ myeloid DCs (1 × 10^5^) were purified to 99.8% purity by flow cytometric sorting and exposed to polyI:C (25 μg/ml), EBV (MOI of 1, 5 × 10^5^ RIU/ml), and LPS in the absence and presence of polymyxin B (25 μg/ml), an inhibitor of LPS-mediated TLR4 activation. IL-12p70 was detected by ELISA 24 h later. (B) Purified DCs were exposed to polyI:C (25 μg/ml) or EBV (MOI of 1, 5 × 10^5^ RIU/m), and upregulation of the DC maturation marker CD83 was detected by flow cytometry 24 h later. (C) Flow-sorted CD11c^+^ DCs and peripheral blood NK cells were cultured together or separately in the presence of polyI:C (25 μg/ml), or EBV (MOI of 1, 5 × 10^5^ RIU/ml). IFN-γ was detected by ELISA 24 h later. Where indicated, IL-12 was blocked in selected experiments with a specific antibody (n.d., not determined). Data represent results from at least three independent experiments performed in duplicates.

In order to extend our findings from monocyte-derived DCs to human blood DCs and from polyI:C to maturation by EBV, we exposed sorted human CD11c^+^ myeloid DCs to EBV particles directly (DC:EBV MOI = 1:1). We observed 189 ± 20 pg/ml IL-12 secretion and upregulation of the maturation marker CD83 upon coculture of myeloid DCs with EBV (Figure 5A and 5B). Both infectious and heat-inactivated EBV elicited this DC maturation (Figure 5A). DC maturation by EBV was not due to endotoxin contamination of the EBV virus preparations, since we detected less than in 0.1 ng endotoxin in 1 × 10^5^ EBV RIU, a concentration insufficient for human DC maturation (Figure 5A). Furthermore polymyxin B, which inhibits TLR4 stimulation by LPS [36], had no effect on EBV-mediated DC maturation, but significantly inhibited DC maturation by LPS (Figure 5A). While EBV induced IL-12 levels were lower than IL-12 concentrations in response to polyI:C and to high levels of LPS (1286 ± 188 pg/ml and 763 ± 87 pg/ml, respectively; Figure 5A), EBV-matured DCs stimulated purified autologous NK cells to secrete IFN-γ in excess of 4000 pg/ml via IL-12 (Figure 5C). These IFN-γ concentrations are high enough to inhibit B cell transformation by EBV in vitro (Figure 4E). These data suggest that human myeloid DCs can be matured by EBV and then activate NK cells to produce protective amounts of IFN-γ.

### IFN-γ Regulates B Cell Transforming EBV Latency

**Figure 6:**
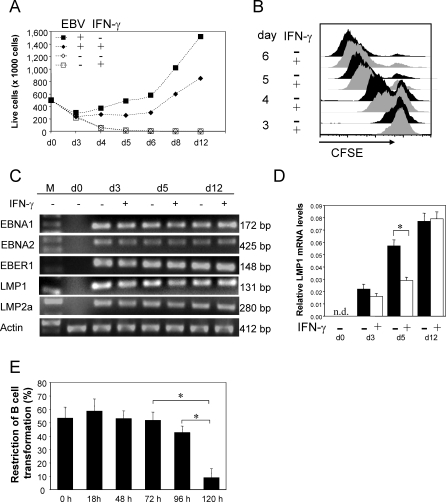
IFN-γ Impairs Transformation of B Cells by EBV (A) Cell numbers were compared at different time points between control and IFN-γ-treated B cells with and without EBV infection. (B) CFSE-labeled B cells were infected with EBV, and proliferation was compared between controls and IFN-γ-treated samples at the indicated time points. (C) Expression of EBV-encoded genes was quantified by RT-PCR at different time points and compared between controls and IFN-γ-treated cells. M, 100 bp ladder (D) Expression of the oncogene LMP-1 was quantified by real time-PCR and normalized to GAPDH expression. (E) Peripheral blood B cells were infected with EBV, and 10,000 pg/ml IFN-γ was added at the indicated time points. Restriction of B cell transformation was analyzed after 12 d by comparing numbers of transformed B cells with and without IFN-γ. Results represent data from at least three independent experiments.

In order to investigate how IFN-γ restricts B cell transformation, we infected B cells with EBV and compared total cell numbers, proliferation and expression of EBV-encoded genes between untreated and IFN-γ-treated cells. We started observing significant differences in cell numbers from day 4 (Figure 6A). This coincided with beginning EBV-infected B cell proliferation, which was delayed when cells were treated with IFN-γ (Figure 6B). Comparing expression of different EBV-encoded genes showed that EBER1, EBNA2, and EBNA1 were similarly up-regulated in untreated and IFN-γ-treated EBV-infected B cells. In contrast, we observed a delayed up-regulation of LMP1 in IFN-γ-treated cultures (Figure 6C and data not shown). Quantitative RT-PCR demonstrated that expression of LMP1 was reduced by 28% and 49% at days 3 and 5 post infection, while at later stages similar LMP1 levels were observed with and without IFN-γ addition, when normalized to GAPDH (Figure 6D). Notably, recombinant IFN-γ did not mediate restriction of B cell transformation when added later than 96h after EBV infection (Figure 6E), and also did not inhibit growth of established EBV-transformed B cell lines (data not shown). We did not observe any effect of IFN-γ on B cell viability as measured by counting live cells up to day 6 post-infection, and levels of the EBV receptor CD21 were not affected by IFN-γ treatment (data not shown). Therefore, we suggest that DC activated NK cells limit B cell transformation by EBV via regulation of EBV latent infection, at least in part via delaying LMP1 expression via IFN-γ.

## Discussion

NK cells and DCs are central figures in the innate immune response, and have been shown to interact in early phases of murine herpes virus infections [18,19,34,37]. In contrast to the mouse, humans possess the CD56^bright^CD16^−^ subset of NK cells, which rapidly secretes high IFN-γ levels and strongly proliferates upon activation by DCs [29]. These NK cells are enriched in secondary lymphoid organs like tonsils and lymph nodes, and are therefore strategically positioned to rapidly respond to pathogens at these sites [26–28]. Such a pathogen is the human tumor virus EBV, which enters the human body through the tonsils after transmission via saliva exchange. Within tonsils of healthy virus carriers, the proliferation program, which is also observed in in vitro EBV-infected B cells, was found in naïve B cells, which travel through the perifollicular T cell zone and follicular mantle zone to encounter antigen [38,39]. These areas were also described to harbor or are close to DC/NK cell interactions [30,40,41]. Our data suggest that at these sites, human DCs can activate preferentially CD56^bright^CD16^−^ NK cells, which then become able to limit EBV-mediated B cell transformation, mainly by secretion of IFN-γ, and regulate the proliferation program of EBV latency via this cytokine. Restriction of EBV-induced B cell transformation by NK cells probably curtails EBV infection until it can be efficiently immune controlled by the adaptive immune system. These results suggest for the first time an important effector function for tonsilar NK cells early in the primary immune response against human persistent and oncogenic EBV.

iDCs patrol the periphery and act as sentinels for the immune system [42]. Upon direct infection by a pathogen or uptake of pathogen-containing material in conjunction with a maturation stimulus they migrate to secondary lymphoid organs carrying information both in the form of a particular maturation pattern and pathogen constituents. DC maturation changes drastically the properties of DCs converting them into potent activators of both the innate and adaptive immune system. One group of DC receptors that detect pathogenic determinants and trigger the activating functions of DCs are TLRs [43]. In murine herpes virus infections, it has been shown that different pathways synergize for the activation of immune responses against these pathogens. TLR9-deficient mice as well as TLR3- and TLR2-deficient mice have increased MCMV titers, suggesting that the immune system uses complementing recognition systems in herpes virus infection [18,20,44]. Similarly, EBV might activate human DCs by means of several pathways. In addition to TLR9-activating CpG-motifs, by which EBV activates human plasmacytoid DCs [45], the EBV genome supports convergent transcription, which occurs also in other DNA viruses such as herpes simplex virus-1 (HSV-1) [46,47]. The resulting virally encoded dsRNAs have been isolated from HSV-1-infected cells [48]. Indeed, dsRNA from the convergently transcribed LMP1 and LMP2A antigens of EBV was able to stimulate IL-12 secretion. The measured IL-12 amounts were similar to levels secreted by CD11c^+^ DCs after exposure to EBV particles (Figure 5A; Table 1). Consequently, we suggest that myeloid DCs can detect EBV either directly, or indirectly through EBV-derived dsRNA as TLR3 and mda-5 agonists during primary infection and subsequently initiate the immune response by activating NK cells and priming of T cells [49,50]. Along these lines, DC-activated NK cells might have limited B cell transformation by EBV in a previous study from our lab, but complete regression of transformed B cells was only achieved after DC dependent priming of EBV-specific T cell responses and was also observed with purified CD4^+^ and CD8^+^ T cells [51].

In line with previous studies, we find that phenotypical markers such as MHC class II and costimulatory molecules are equally upregulated with various DC maturation stimuli, while cytokine secretion profiles varied dramatically between different DC maturation conditions. In particular, IL-12p70, a potent stimulator of NK cells and Th1 responses, was produced at higher levels by DCs exposed to EBV, or matured with EBV-derived dsRNA or with maturation cocktails containing the dsRNA analog polyI:C, compared to immature DCs or DCs matured with proinflammatory cytokines or LPS. Nonetheless, even low levels of IL-12 secreted by LPS-matured DCs through directed secretion into the synapse between NK cells and DCs have been shown to activate NK cells [52]. Hence, our data suggest that myeloid DCs stimulate NK cell responses during primary EBV infection at least in part via IL-12.

Two main functional subsets of NK cells have been described in humans, while counterparts for these NK cell populations have not been identified in the mouse so far [25]. The CD56^dim^CD16^+^ subset is mainly responsible for natural cytotoxicity and antibody-dependent cell mediated cytotoxicity (ADCC), while the CD56^bright^CD16^−^ subset has been characterized by its unique capacity to produce high amounts of immunoregulatory cytokines, such as TNF, IFN-γ and GM-CSF, upon activation [25]. IFN-γ production by CD56^bright^CD16^−^ NK cells, as well as NK cell proliferation are rapidly induced by DCs [30]. In addition, DCs also augment cytotoxicity of this subset after prolonged activation [53]. Several studies indicate a role for NK cells in the control of EBV infection and in particular in early primary immune responses [8–10,54]. However, all in vitro studies up to now have used blood NK cells activated by IL-2, which is, at least in humans, mostly secreted by activated T cells and therefore presumably not present during innate immune responses. Hence, we focused on NK activation by DCs as a physiological NK cell stimulus present during the early immune response to primary virus infections. Furthermore, previous studies on the role of NK cells during EBV infection mainly emphasized cytotoxicity, but not IFN-γ secretion of NK cells, although lymph node- and tonsil-resident NK cells primarily release cytokines after activation [26,28,30]. Suggesting a prominent role for NK cell-derived cytokines early during EBV infection, earlier studies have found that recombinant IFN-γ is protective for several days during initial B cell transformation by EBV, whereas in contrast, type I interferons inhibit transformation only during the first hours after infection [55]. Moreover, EBV-specific CD4^+^ and CD8^+^ T cells have been reported to mediate regression of EBV-transformed B cells despite low to undetectable cytolytic activity [56,57]. Our data support the hypothesis that tonsilar NK cells restrict efficiently EBV-induced B cell transformation via their superior ability to produce IFN-γ upon DC activation. Although IL-12 and IFN-α secreted by polyI:C DCs and DC1s upregulate cytotoxicity of NK cells (Figure S3), autologous EBV-transformed B cells are not efficiently killed by activated NK cells due to their high MHC class I expression (data not shown and [12]). Instead, the results of this study support a prominent role for IFN-γ in the innate immune response to EBV by NK cells. First, addition of recombinant IFN-γ to the B cell transformation assay decreased the number of transformed B cells. Second, IFN-γ levels, sufficient to restrict EBV-induced B cell transformation, were found in our cocultures of EBV-infected B cells with DCs and NK cells. Finally, blocking of IFN-γ in B cell transformation assays with NK cells significantly decreased the protective effect of NK cells. Hence, we conclude that this cytokine significantly contributes to innate resistance against primary EBV infection. In addition to its direct antiviral activity, IFN-γ secreted by DC-activated NK cells might also shape the EBV-specific adaptive immune response favoring a Th1-polarization which is observed in EBV-positive individuals [58–61]. Therefore, contrary to the original paradigm that NK cells respond primarily with cytotoxicity without prior activation, which gave this lymphocyte subset its name, we demonstrate that their main function against a relevant human pathogen consists of cytokine secretion after activation by DCs.

EBV transforms B cells by the coordinate expression of EBV latency genes that provide signals for B cell survival and proliferation. Of the eight latent EBV antigens, LMP1 has been suggested to be the main oncogene of the virus, causing epithelial cell transformation in vitro and B cell transformation in vivo [62–64]. Therefore, IFN-γ induced down-regulation of LMP1 transcription could be one mechanism by which DC-activated NK cells limit EBV-induced B cell expansion. The fairly late transcription of LMP1, compared to other EBV latent antigens, during the establishment of EBV latency could also explain why IFN-γ can restrict B cell transformation by EBV during the first days of primary EBV infection, while others and we found that IFN-γ was not able to inhibit proliferation of fully EBV-transformed lymphoblastoid cell lines (LCLs) ([55] and data not shown). Similarly, IFN-γ secretion by NK cells was shown to limit MCMV infection during the first week of infection, and reduced immediate early or late MCMV gene transcription, depending on the infected cell type [33,65]. Therefore, tonsilar NK cells might limit latent EBV infection by IFN-γ mediated down-regulation of LMP1 until adaptive T cell immune responses can eliminate fully EBV-transformed B cells.

In summary, we suggest that myeloid DCs stimulate NK cells during EBV infection primarily via their ability to secrete IL-12. Activated NK cells are then able to mediate restriction of EBV-mediated B cell transformation. Tonsilar NK cells, which, like lymph node NK cells, produce higher levels of IFN-γ than their peripheral blood counterparts after activation by DCs, are superior in inhibiting EBV-induced B cell transformation in vitro by down-regulating important components in the proliferation program of EBV latency. These results suggest a novel and important effector function for tonsilar CD56^bright^CD16^−^ NK cells upon DC activation in the primary immune response against EBV. Beyond EBV infection, our data suggest that humans have a strategically well-positioned population of NK cells that directly combats pathogen entry at mucosal sites and might restrict pathogens until they can be cleared or controlled by adaptive immunity.

## Materials and Methods

### Antibodies.

The following directly-labeled monoclonal antibodies were used for flow cytometry: anti-CD3 (clone SK7), anti-CD11c (B-ly6), anti-CD19 (HIB19), anti-CD21 (B-ly4), anti-CD23 (M-L233), anti-CD56 (B-159), anti-CD80 (L307.4), anti-CD86 (IT2.2), anti-HLA-DR (TU36), anti-IFN-γ (B27, all BD Biosciences), anti-CD16 (3G8, Caltag), anti-CD25 (PC61 5.3), anti-CD83 (HB15a, both Beckman Coulter), and anti-IL-15 (34599, R&D Systems). Cells labeled with goat-polyclonal anti-IL-15Rα (R&D Systems) were stained with AlexaFluor-488 rabbit-anti-goat-IgG (Molecular Probes). The following monoclonal antibodies were used for antibody-mediated blocking: anti-IL-12 (clone 24910), anti-IL15 (34593, both R&D Systems), and anti-IL-18 (125–2H, MBL International). IgG1 (MOPC-21, BioLegend) was used as control.

### Tonsils, lymph nodes, and spleens.

All tonsils, lymph nodes and spleens were obtained as part of Institutional Review Board-approved protocols. Tonsils were collected immediately after surgery from patients undergoing tonsilectomy for chronic inflammation. Tonsils were not acutely inflamed at the time of removal. Spleens and lymph nodes were procured by the regional Organ Procurement Organization from brain-dead donors after obtaining informed consent from appropriate individuals. Soon after their removal, tissues were mechanically dissociated to obtain single cell suspensions and were then filtered through a 75-μm nylon cell strainer to exclude undissociated fragments. Debris and dead cells were eliminated using Ficoll/Hypaque (Amersham Pharmacia) discontinuous gradient centrifugation. Single cell suspensions were then extensively washed and cryopreserved.

### Preparation of DCs.

PBMCs were isolated from leukocyte concentrates (New York Blood Center) by density-gradient centrifugation on Ficoll/Hypaque. CD14^+^ cells were isolated from PBMCs by positive magnetic cell separation (MACS, Miltenyi Biotec) and cultured for 5 days in RPMI1640 + 1% single donor plasma + IL-4 and GM-CSF according to standard protocols [30]. The CD14^−^ cells were frozen for later isolation of B cells and NK cells. Splenic DCs were isolated as previously described by flow cytometric sorting using a BD FACSVantage SE cell sorter [30]. To isolate CD11c^+^ cells from blood, PBMCs were overlayed with an Optiprep gradient (1.080 to 1.049) and centrifuged for 30 min at 700×g. Low-density fractions were collected and CD11c^+^ DC were further enriched by depletion of CD14^+^, CD3^+^, CD8^+^, and CD19^+^ cells by MACS. CD11c^+^ DCs were purified by flow cytometric sorting using a BD FACS Aria cell sorter by isolating lin- (CD3, CD14, CD19, and CD56), HLA-DR^+^, and CD11c^+^ cells. Purify after sorting was regularly higher than 99.5%. DCs were matured for 2d in medium with IL-4, GM-CSF, and i) 10 ng/ml IL-1β, 1,000 units/ml IL-6, 10 ng/ml TNF-α, and 1 μg/ml prostaglandin E_2_ (cyt DC), ii) 25 μg/ml polyinosine-polycytidylic acid (polyI:C, Invivogen) (polyI:C DC), iii) 25 ng/ml IL-1β, 50 ng/ml TNF-α, 3,000 IU/ml IFN-α, 500 pg/ml IFN-γ, and 25 μg/ml polyI:C (DC1), iv) 250 ng/ml LPS (Sigma) (lps DC). CD11c^+^ DCs were exposed to AGS-cell derived EBV at an MOI of 1. Maturation of DCs was monitored by flow cytometry using anti-CD25, anti-CD80, anti-CD83, anti-CD86, and anti-HLA-DR. Secretion of cytokines was quantified using IL-12p70 ELISA, IL-15 ELISA (both R&D Systems) and IL-18 ELISA (Bender Medsystems).

### Isolation of B cells and NK cells.

Frozen CD14^−^ PBMCs were thawed, washed and B cells were isolated by positive selection using CD19^−^ Microbeads (Miltenyi Biotec). NK cells were isolated from either CD19^−^ or CD14^−^ fractions by negative selection using the NK cell Isolation Kit II (Miltenyi Biotec) according to the manufacturer's instructions. The purity of the isolated B cells and NK cells was higher than 90% and contained less than 5% contaminating T cells as determined by flow cytometry. For other experiments, B cells, NK cells, and NK cell subsets were isolated by flow cytometric sorting using a BD FACSVantage SE cell sorter.

### Preparation of EBV.

The EBV^+^ marmorset cell line B95–8 was seeded at 2 × 10^5^ cells / ml and cultured for 12 d in RPMI1640 + 10% FCS + gentamycin without refeeding. Virus-containing supernatant was centrifuged at 2000 rpm for 10 min and passed through a 0.45 μm filter. EBV^+^ AGS cells were used to produce EBV as previously described, and virus was further concentrated and purified by ultracentrifugation [66]. AGS-derived EBV preparations contained less than 0.1 EU/ml endotoxins as measured by LAL test (Cambrex Corporation).

### Generation of EBV-derived dsRNA.

A 1.3 kB fragment spanning the coding region of LMP1 in one direction and part of the first intron of LMP2A in the other direction was cloned into pGEM (Promega) between the T7 and SP6 promoter. ssRNA was generated using linearized plasmid and the Riboprobe Combination System SP6/T7 (Promega). After verification of integrity of RNA by gel electrophoresis, ssRNA was purified using RNeasy Kit (Qiagen) and quantified by Nanodrop. Finally, equal amounts were annealed in siRNA buffer (Dharmacon) to generate dsRNA and successful annealing was confirmed by gel electrophoresis.

### Proliferation assay.

Isolated NK cells were labeled with 1 μM CFSE in PBS plus 0.1% BSA for 10 min at 37°C. After washing twice with RPMI1640 + 5% human serum + gentamycin, NK cells (2.5 × 10^5^ cells in 96 well plate) were cultured with 500 IU/ml IL-2 or autologous DCs at a ratio of 5:1 for 6 days at 37°C in RPMI1640 + 5% human serum + gentamycin. In selected experiments, isotype control antibody (5 μg/ml) or blocking antibodies against IL-12, IL-15 (5 μg/ml each), and IL-18 (1 μg/ml) were added to the cultures at the beginning and on day 3 of culture. Where indicated, DCs (bottom) were separated from NK cells (top) by 0.4 μm pore membranes (Corning). CFSE fluorescence and CD16 staining was evaluated on CD3^−^CD56^+^ cells by flow cytometry. Where indicated, B cells were CFSE labeled as described above and proliferation of CD19^+^CD20^+^ cells was evaluated with and without 10,000 pg/ml IFN-γ addition after infection with EBV as CFSE dilution by flow cytometry. Live cell numbers were determined by trypan blue exclusion.

### IFN-γ assay.

For intracellular staining of IFN-γ, isolated NK cells from blood (2.5 × 10^5^ cells in 96 well plate) were incubated with 500 IU/ml IL-2 or autologous DCs at a ratio of 1:2 at 37°C in RPMI1640 + 5% human serum + gentamycin. In selected experiments, isotype control antibody (5 μg/ml) or blocking antibodies against IL-12, IL-15 (5 μg/ml each), and IL-18 (1 μg/ml) were added. Brefeldin-A was added after 6 h of coculture and additional 6 h later cells were harvested and stained with anti-CD3, anti-CD56 and anti-CD16. After fixing the cells with 2% paraformaldehyde, they were permeabilized and stained with anti-IFN-γ. In other experiments, DCs (bottom) were separated from NK cells (top) by 0.4 μm pore membranes and IFN-γ production was compared to NK-DC cocultures without separation after 20h using an IFN-γ ELISA (Mabtech). To directly compare IFN-γ production, sorted NK cell subsets from blood, spleen, tonsil and lymph node (1 × 10^4^ cells CD56^bright^CD16^−^, 1 × 10^5^ CD56^+^CD16^+^) were then cultured with allogeneic or autologous DCs at a ratio of 1:10 or 1:1, respectively. After 20 h IFN-γ levels were determined using ELISA.

### B cell transformation assay with peripheral blood, splenic, and tonsilar mononuclear cells.

Isolated B cells (1 × 10^5^ cells in 48 well plate) were cultured in RPMI1640 + 5% human serum + gentamycin, infected with EBV and isolated NK cells were added at indicated numbers. In experiments including DCs, they were added at B cell to DC ratios of 1:1. In other experiments, B cells and DCs (bottom) were separated from NK cells (top) by 0.4 μm porous membranes. After 12 d, numbers of transformed B cells were quantified by counting live cells via trypan blue exclusion and determining the ratio of CD19^+^CD21^+^CD23^+^ cells to total live cells by flow cytometry. Restriction of B cell transformation was calculated by comparing numbers of transformed B cells between respective samples with and without NK cells; % Restriction of B cell transformation = (1 − total transformed B cell number of sample with NK cells/total transformed B cell number of sample without NK cells) × 100.

### B cell transformation assay with bulk tonsilar mononuclear cells.

Cryopreserved tonsilar mononuclear cells were thawed, washed, and then stained with anti-CD3 and anti-CD56. Cells were then depleted (i) of CD3^+^ cells (Tonsil -T) or (ii) of CD3^+^ and CD56^+^ cells (Tonsil -NK-T) by flow cytometric sorting using a BD FACSVantage SE cell sorter. The number of sorted cells per condition was adjusted according to the ratio between sorted cells and input cell numbers (1 × 10^6^ cells in 48 well plate). Then, the cells were infected with EBV and, where indicated, DCs were added at ratio of total cell to DC of 10:1. After 12 d, numbers of transformed B cells were quantified by counting live cells via trypan blue exclusion and determining the ratio of CD19^+^CD21^+^CD23^+^ cells to total live cells by flow cytometry. Restriction of B cell transformation was calculated by comparing numbers of transformed B cells between respective samples with and without NK cells; % Restriction of B cell transformation = (1 − total transformed B cell number of sample with NK cells/total transformed B cell number of sample without NK cells) × 100.

### Quantification of EBV mRNAs.

RNA was isolated from non-treated and IFN-γ-treated B cells infected with EBV at indicated time point using the RNeasy Kit (Qiagen) according to the manufacturer's instructions. 10 ng of total RNA were used for semi-quantitative RT-PCR using the OneStep RT-PCR-Kit (Qiagen) and gene-specific primers using the following program: 50°C (30 min), 95°C (15 min), followed by 35 cycles of 95°C (30 sec), 55°C (30 sec), and 72°C (30 sec). Primers sequences were as follows: Actin 5′-CAAGAGATGGCCACGGCTGCT, Actin 3′-TCCTTCTGCATCCTGTCGGCA, EBNA1 5′-GAGCGTTTGGGAGAGCTGAT, EBNA1 3′-CATTTCCAGGTCCTGTACCT, EBNA2 5′-CATAGAAGAAGAAGAGGATGAAGA, EBNA2 3′-GTAGGGATTCGAGGGAATTACTGA, EBER1 5′-AAAACATGCGGACCACCAGC, EBER1 3′-AGGACCTACGCTGGCCCTAGA, LMP1 5′-AGGTTGAAAACAAAGGAGGTGACCA, LMP1 3′-GGAACCAGAAGAACCCAAAAGCA, LMP2a 5′-ATGACTCATCTCAACACATA, LMP2a 3′-CATGTTAGGCAAATTGCAAAA. To generate cDNA for RealTime-PCR, 500 ng total RNA were reverse transcribed per 50 μl reaction using the TaqMan Reverse Transcription Reagent (Applied Biosystems), using the following program: 25°C (10 min), followed by 50°C (30 min) and 75°C (5 min sec). Amplification was performed in a final volume of 20 μl, containing 2 μl cDNA from the reversed transcribed reaction, primer mixture (0.25 μM each of sense and antisense primers), and 10 μl of 2× SYBR Green Master Mix (Applied Biosystems). PCR was performed in ABI 7900HT Sequence Detection System (Applied Biosystems) using following program: 95°C (15 min), followed by 40 cycles of 95°C (15 sec), 55°C (30 sec), and 68°C (30 sec). The final mRNA levels of LMP1 were normalized to GAPDH using the comparative C_T_ method. Primers sequences for RealTime-PCR were as follows: GAPDH 5′-AGCCACATCGCTCAGACAC, GAPDH 3′-GCCCAATACGACCAAATCC, LMP1 5′-AGGTTGAAAACAAAGGAGGTGACCA, LMP1 3′-GGAACCAGAAGAACCCAAAAGCA.

### NK cell cytotoxicity assay.

To evaluate the cytolytic activity after DC activation, NK cells were cocultured for 2 d with DC1s at a ratio of 5:1 with or without blocking antibodies against IL-12 and type I interferon receptor (anti-CD118, clone MMHAR-2, PBL Biomedical Laboratories). The NK cell-sensitive lymphoblastoid cell line LCL 721.221, which does not express surface HLA class I molecules, was used as target cell. Cytotoxicity assays were performed, as previously described [28]. Briefly, target cells were labeled with PKH26 (Sigma-Aldrich, St. Louis, MO), and then incubated with NK cells at different NK cell/target cell ratios. After 6 h, cells were harvested; TO-PRO-3, a membrane-impermeable DNA stain, was added to each culture (1 μM final concentration); and cells were finally analyzed by flow cytometry. Background and maximum TO-PRO-3 staining were obtained by incubation of target cells with medium and detergent, respectively. The percent specific lysis was calculated as (% TO-PRO-3^+^PKH26^+^ cells in NK/target cell co-culture − % TO-PRO-3^+^PKH26^+^ cells in medium)/(% TO-PRO-3^+^PKH26^+^ cells in detergent − % TO-PRO-3^+^PKH26^+^ cells in medium) × 100%.

### Statistical analysis.

Statistical analyses were performed with the paired two-tailed Student t-test. The *p*-value of significant differences is reported. Plotted data represent mean plus standard deviation (SD), unless otherwise stated.

## Supporting Information

Figure S1 NK Activation by PolyI:C-matured DCs Is IL-12 Dependent and Is Mainly Restricted to CD56^bright^CD16^−^ NK Cells (158 KB PDF)

Figure S2 DC1 Induced NK Cell Proliferation and IFN-γ Production Is IL-2 Independent (110 KB PDF)

Figure S3 Upregulation of NK Cell Cytotxicity by DCs Depends on IL-12 and Type I Interferons (81 KB PDF)

Figure S4 Tonsilar NK Cells Do Not Express CCR7 and Only a Low Number Expresses CD83 (74 KB PDF)

Table S1 Surface Expression of DC Maturation Markers (74 KB PDF)

### Accession Numbers

Accession numbers mentioned in this study are from Swiss-Prot (http://www.ebi.ac.uk/swissprot/) and from GenBank (http://www.ncbi.nlm.nih.gov/Genbank/), respectively: hIL-2, P60568; hIL-12p35, P29459; hIL-12p40, P29460; hIL-15, P40933; hIL-15Rα, Q13261; hIL-18, Q14116; hIFN-γ, P01579; hIFNα/β-R, P17181; hCD3, P07766; hCD16, P08637; hCD25, P01589; hCD56, P13591; hCD80, P33681; hCD83, Q01151; hCD86, P42081; EBNA1, P03211; EBNA2, P12978; LMP1, P03230; LMP2a, P13285; EBER1, J02078; hβ-actin, P60709; hGAPDH, P04406.
