# Supplementary material for: Tonsilar NK Cells Restrict B Cell Transformation by the Epstein-Barr Virus via IFN-γ
Source: PLoS Pathog. 2008 Feb 8;4(2):e27. doi: 10.1371/journal.ppat.0040027 (PMC2233668; doi:10.1371/journal.ppat.0040027)
Supplement: Supplementary file 1 [file ppat.0040027.sg001.pdf]

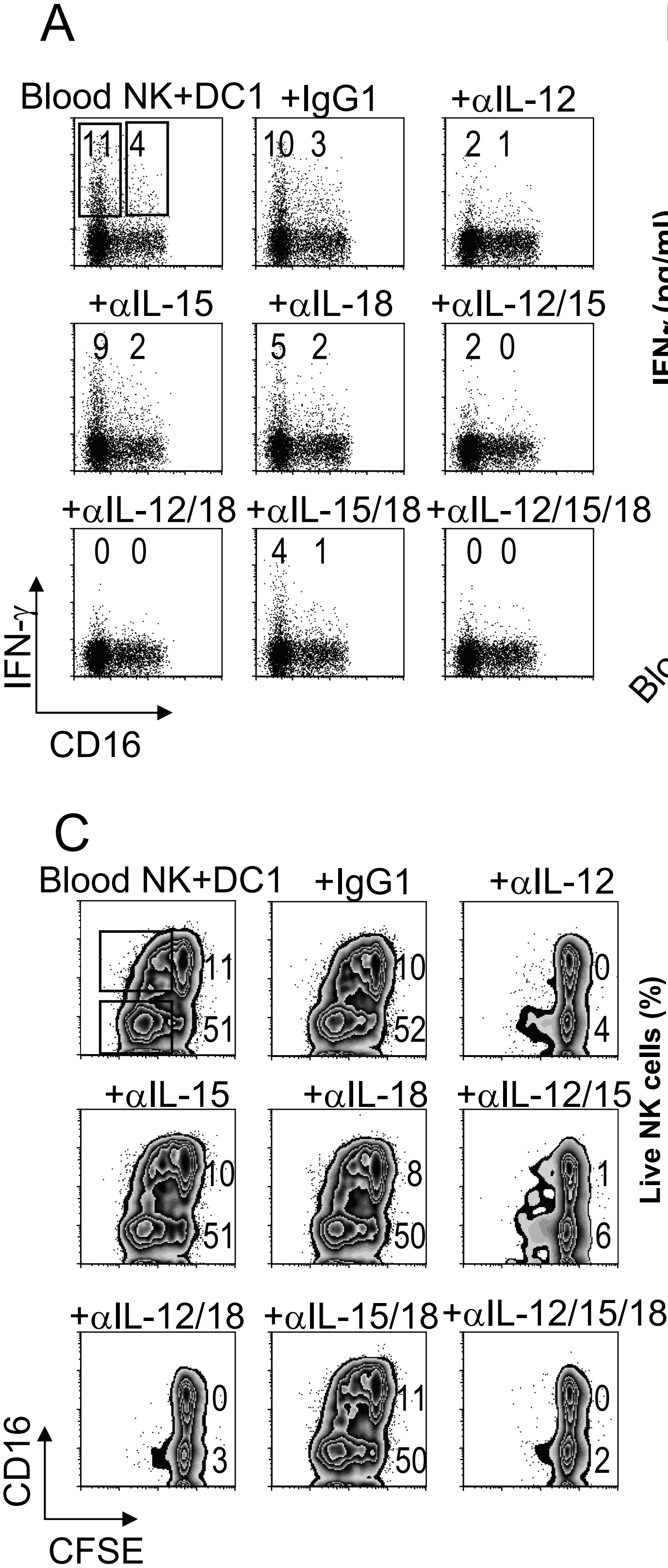

Figure S1: NK activation by polyI:C-matured DCs is IL-12 dependent and is mainly restricted to CD56[bright]CD16[-] NK cells. (A) Blood NK cells were activated by DC1s in the presence or absence of blocking antibodies and IFN-γ production of CD3[-]CD56[+] cells was assayed by intracellular cytokine staining. Percentages of IFN-γ positive NK cells are indicated. (B) NK cells were cultured directly or separated by transwell with DC1s. In addition, cytokines were blocked in transwell experiments using blocking antibodies. IFN-γ levels were measured by ELISA (mean ± s.d.) (C) NK cells were cultured with DC1s in the presence or absence of blocking antibodies for 6 d and CFSE dilution of CD3[-]CD56[+] cells was analyzed. Percentages of CFSE dilute CD16[+] and CD16[-] NK cells are indicated. (D) After 6 d of NK cell-DC cocultures, live cells were counted and subsequently, numbers of total and surviving CD3[-]CD56[+] NK cells were determined by measuring ratios of total and proliferating NK cells of total live cells. Data represents numbers of proliferating and total NK cells compared to controls without antibody blocking (mean ± s.d.). Mouse-IgG1 was used in all experiments as isotype control. Data in (A)-(D) represent results of at least three independent experiments.
