# Supplementary material for: Tonsilar NK Cells Restrict B Cell Transformation by the Epstein-Barr Virus via IFN-γ
Source: PLoS Pathog. 2008 Feb 8;4(2):e27. doi: 10.1371/journal.ppat.0040027 (PMC2233668; doi:10.1371/journal.ppat.0040027)
Supplement: Supplementary file 2 [file ppat.0040027.sg002.pdf]

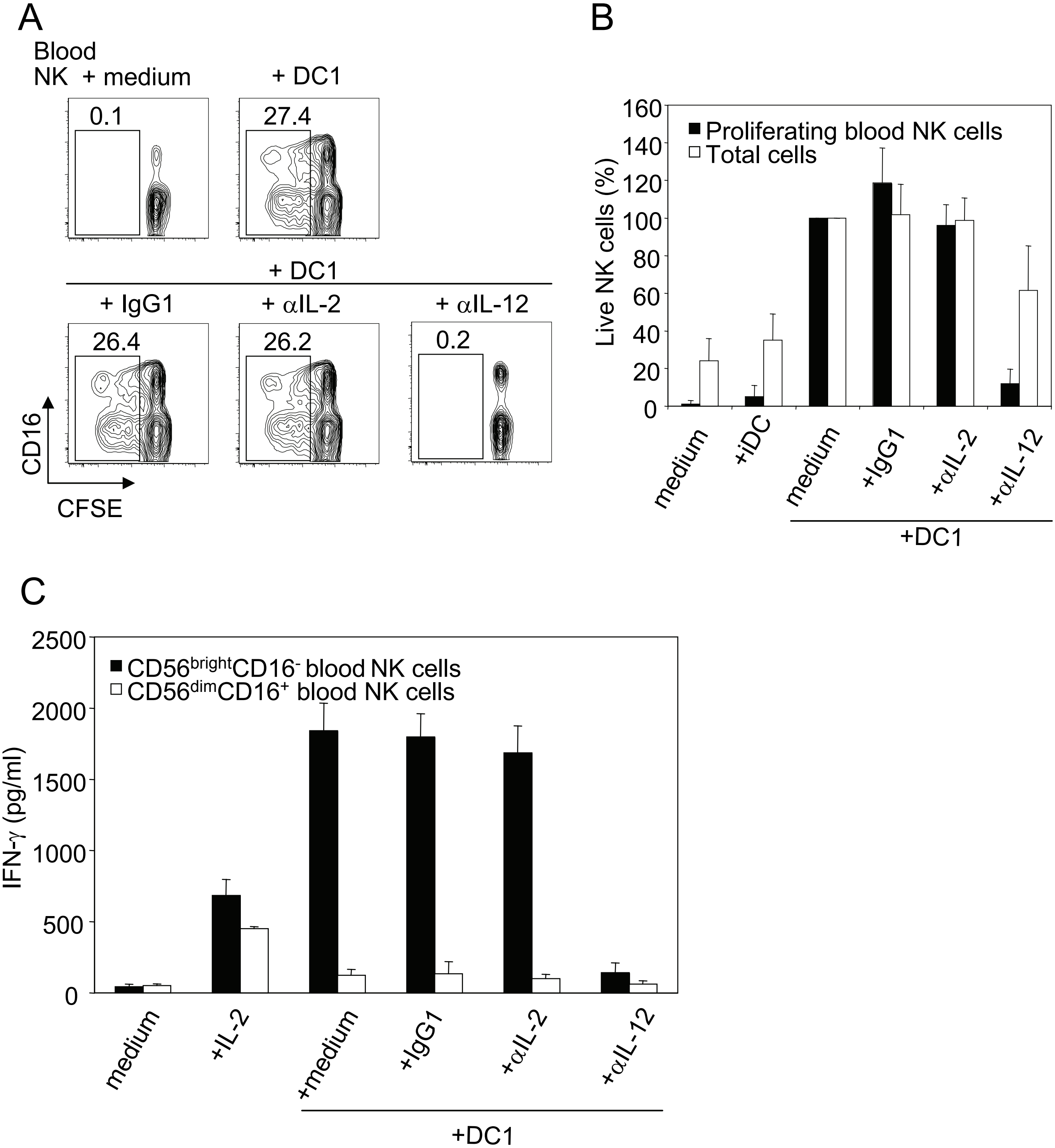

Figure S2: DC1 induced NK cell proliferation and IFN-γ production is IL-2 independent. (A) Blood NK cells were cultured with iDCs or DC1s in the presence or absence of blocking antibodies for 6 d and CFSE dilution of $CD3^{-}CD56^{+}$ cells was analyzed. Percentages of CFSE-dilute NK cells are indicated. (B) After 6 d of NK cell-DC cocultures, live cells were counted and subsequently, numbers of total and surviving $CD3^{-}CD56^{+}$ NK cells were determined by measuring ratios of total and proliferating NK cells of total live cells. Data represents numbers of proliferating and total NK cells compared to DC1 coculture without antibody blocking (mean ± s.d.). Data in (A) and (B) represent results of three independent experiments. (C) Sorted NK cell subsets were cultured with medium, IL-2 (500 U/ml) or DC1s in the presence or absence of blocking antibodies for 20 h and IFN-γ was analyzed by ELISA (mean ± s.d.). Data in (C) represent results of two independent experiments done in duplicates. Mouse-IgG1 was used in all experiments as isotype control.
