# Supplementary material for: Tonsilar NK Cells Restrict B Cell Transformation by the Epstein-Barr Virus via IFN-γ
Source: PLoS Pathog. 2008 Feb 8;4(2):e27. doi: 10.1371/journal.ppat.0040027 (PMC2233668; doi:10.1371/journal.ppat.0040027)
Supplement: Supplementary file 3 [file ppat.0040027.sg003.pdf]

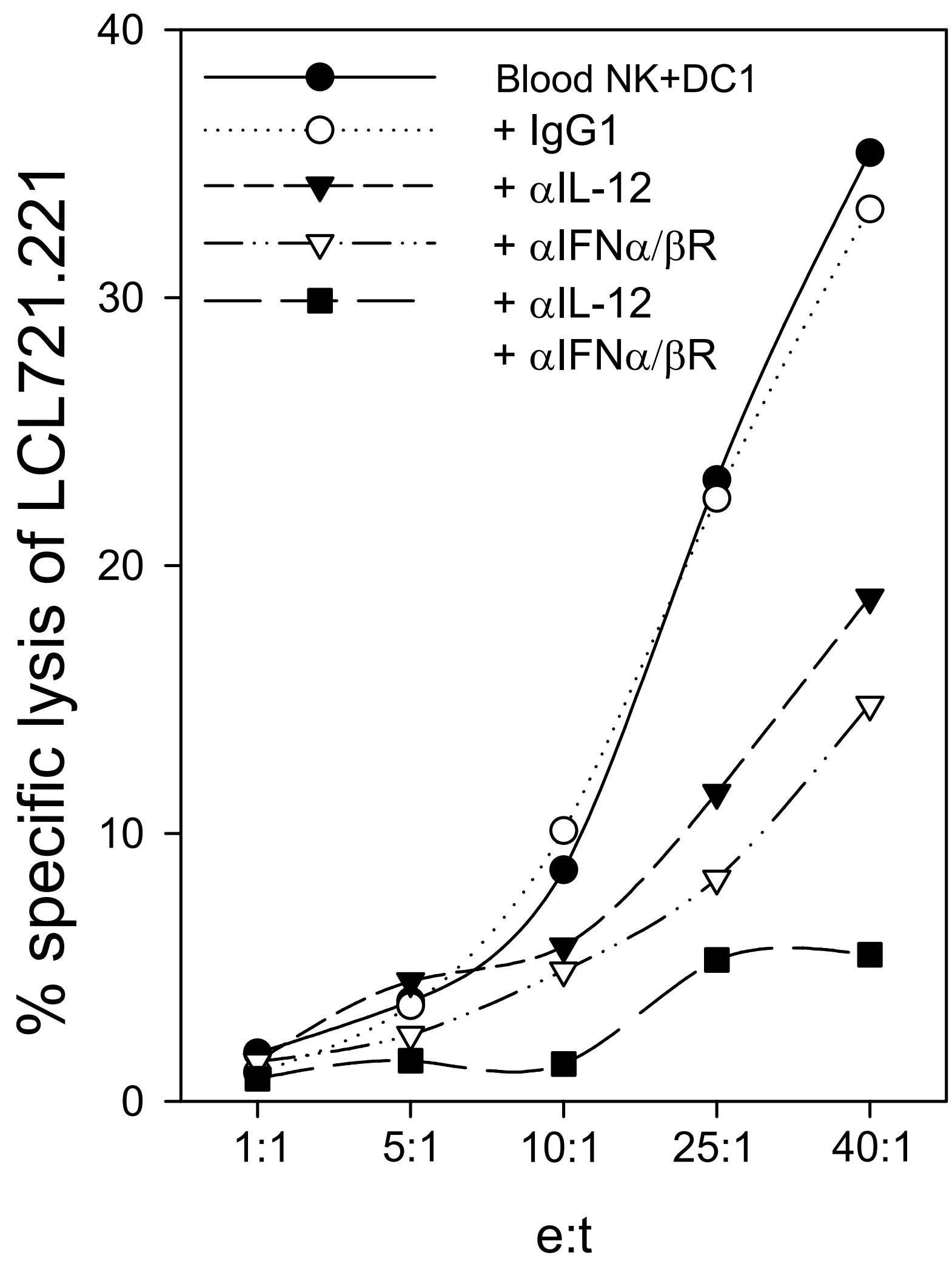

Figure S3: Upregulation of NK cell cytotxicity by DCs depends on IL-12 and type I interferons. Blood NK cells were cocultured with DC1s for 2 d in the presence or absence of blocking antibodies. Upregulation of cytotxicity was assessed by measuring lysis of the NK sensitive target cell line LCL721.221 at the indicated effector to target cell ratios.
