# Supplementary material for: Tonsilar NK Cells Restrict B Cell Transformation by the Epstein-Barr Virus via IFN-γ
Source: PLoS Pathog. 2008 Feb 8;4(2):e27. doi: 10.1371/journal.ppat.0040027 (PMC2233668; doi:10.1371/journal.ppat.0040027)
Supplement: Supplementary file 4 [file ppat.0040027.sg004.pdf]

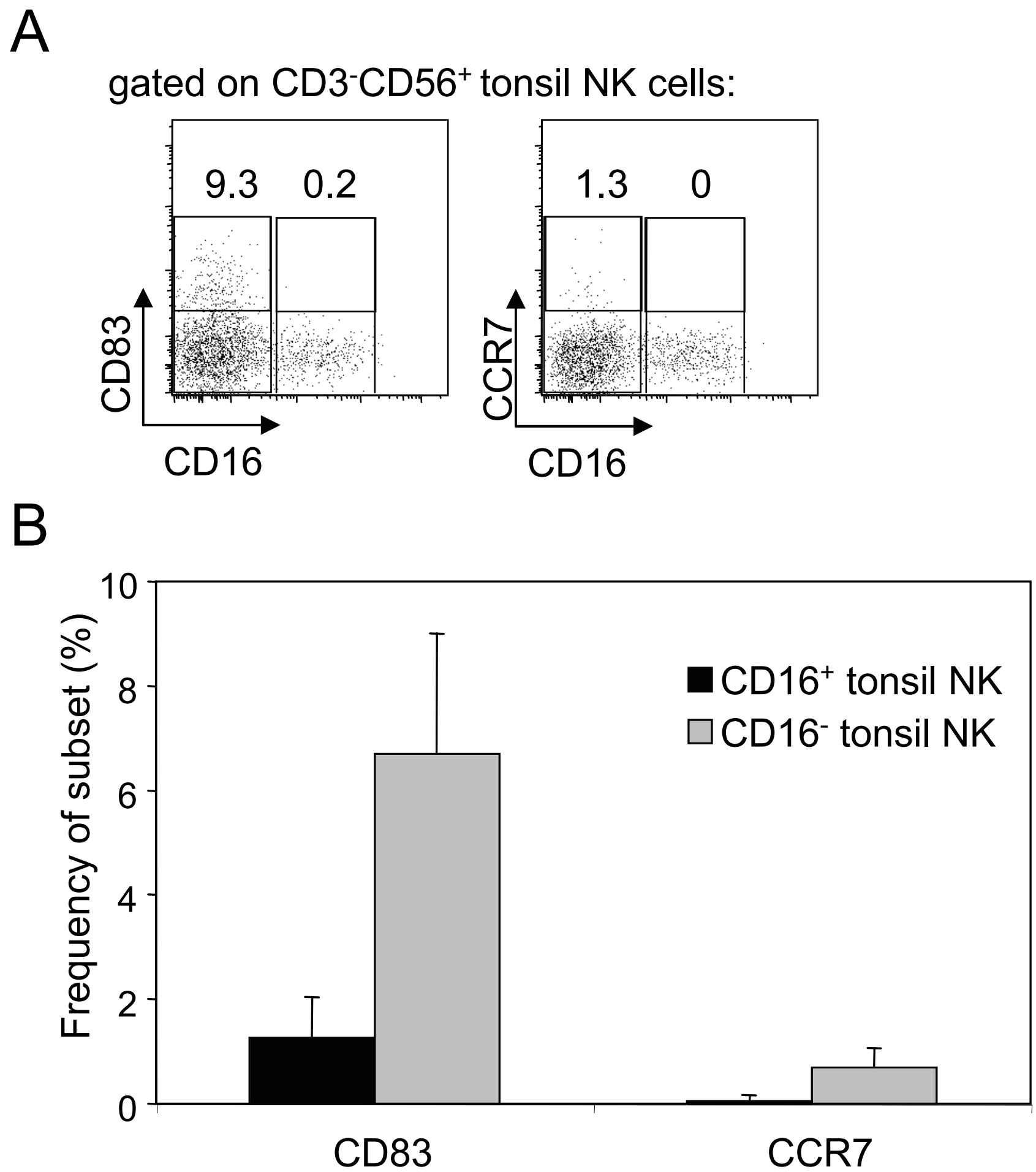

Figure S4: Tonsilar NK cells do not express CCR7 and only a low number expresses CD83. Mononuclear cells from tonsil were stained with CD3 and CD56 to identify $CD3^{-}CD56^{+}$ NK cells. In addition cells were stained with antibodies against CD16 and CD83 or CCR7. (A) shows a representative experiment, and (B) summarizes data from four independent experiments (mean ± s.d.).
