# Supplementary material for: Tonsilar NK Cells Restrict B Cell Transformation by the Epstein-Barr Virus via IFN-γ
Source: PLoS Pathog. 2008 Feb 8;4(2):e27. doi: 10.1371/journal.ppat.0040027 (PMC2233668; doi:10.1371/journal.ppat.0040027)
Supplement: Supplementary file 5 [file ppat.0040027.st001.pdf]

Table S1: Surface expression of DC maturation markers

DC type	maturation	HLA-DR	CD25	CD80	CD83	CD86	IL-15Rα	IL-15
monocyte-derived DCs	none	307[a]	87	301	104	412	4	21
	cyt	435	265	546	407	690	15	62
	polyI:C	429	267	602	390	745	37	75
	DC1	429	347	620	412	742	68	72
	LPS	403	269	558	429	671	20	52
	dsRNA	354	205	450	167	621	8	23

[a]: Values indicate mean flourescence intensities (substracted from isotype control staining)

Similar results were obtained in at least four experiments
